# Perceived barriers to accessing Female Community Health Volunteers’ (FCHV) services among ethnic minority women in Nepal: A qualitative study

**DOI:** 10.1371/journal.pone.0217070

**Published:** 2019-06-10

**Authors:** Sarita Panday, Paul Bissell, Edwin van Teijlingen, Padam Simkhada

**Affiliations:** 1 Walter H. Shorenstein Asia-Pacific Research Centre (APARC), Stanford University, Stanford, California, United States of America; 2 School of Human and Health Sciences, University of Huddersfield, Huddersfield, West Yorkshire, United Kingdom; 3 Faculty of Health & Social Sciences, Bournemouth University, Bournemouth, South West England, United Kingdom; 4 Public Health Institute, Liverpool John Moores University, Liverpool, South West England, United Kingdom; Ben-Gurion University of the Negev Faculty of Health Sciences, ISRAEL

## Abstract

Despite the efforts of community health workers to increase access to healthcare among ethnic minority groups in low- and -middle income countries, members of ethnic minorities are less likely than women from other ethnic groups to use maternal and child healthcare services. However, much less is known about the factors that limit access of ethnic minorities to healthcare services, including the services of community health workers in Nepal, who are known as Female Community Health Volunteers (FCHVs). To address this issue, we conducted a qualitative study to explore perceived barriers to accessing maternal and child healthcare services among ethnic minority groups in two different geographical locations (the hill and Terai regions- flatland bordering south India) with varying degrees of access to local healthcare centres. Between April 2014 and September 2014, semi-structured interviews were conducted with twenty FCHVs, 26 women service users and 11 paid local health workers. In addition, 15 FCHVs participated in four focus group discussions. A thematic analysis of the data identified five major themes underlying barriers to accessing available maternal and child healthcare services by ethnic minority groups such as Dalits, Madhesi, Muslim, Chepang and Tamang. These themes include: a) lack of knowledge among service users; b) lack of trust in volunteers; c) traditional beliefs and healthcare practices; d) low decision-making power of women; and e) perceived indignities experienced when using health centres. We conclude that community health programmes should focus on increasing awareness of healthcare services among ethnic minority groups, and the programmes should involve family members (husband and mothers-in-law) and traditional health practitioners. Both the FCHVs and local healthcare providers should be trained to communicate effectively in order to deliver respectful care among ethnic minorities if we want to achieve universal healthcare coverage for maternal and child health in low- and -middle income countries.

## Introduction

Ethnic minority groups are likely to have worse maternal and child health outcomes and more difficulty in using healthcare than the general population in Nepal [1–3]. To promote women’s use of healthcare, Community Health Workers (CHWs), known as Female Community Health Volunteers (FCHVs), are mobilised across Nepal [4]—a programme which has also been implemented in other Low- and -Middle Income Countries (LMICs) such as India [5], Bangladesh [6], Afghanistan [7] and Uganda [8]. The FCHVs are praised for their contributions to improving maternal and child health [9–11], yet these improvements have been unequal across ethnic groups. Dalit women especially along with indigenous groups such as Chepang and Tamang in the hill regions, and Madhesi and Muslim in the Terai (flatland bordering south of India) are reported to underuse healthcare services despite their availability in local communities [1, 12, 13]. This study sets out to explore perceived barriers to accessing FCHVs’ maternal and child healthcare services among ethnic minority groups in rural Nepal. Below, we briefly introduce the ethnic groups in Nepal and the FCHV programmes relevant to this study.

Nepal is a multi-ethnic (125 ethnic groups) and multilingual (92 spoken languages) country with caste-based hierarchies [14]. The caste-based hierarchies officially began in 1854 when socially, culturally, and linguistically distinct ethnic minorities and indigenous groups were merged to produce five groups. The groups of Bramhin and Kshatri occupied the high caste group and the remaining were classified beneath them with Dalit occupying the bottom of the Hindu caste system. In the Hindu caste system, Dalits are considered untouchable and are widely discriminated against, although caste-based discrimination was officially abolished in 1963 [15]. Similarly, indigenous groups such as Chepang and Tamang in the hill region, and ethnic minorities such as Muslim and Madhesi, in the Terai occupy low social status and continue to underuse healthcare services [3].

In order to address health needs of rural populations, the government of Nepal established the FCHV programme in 1988 [4]. The programme is intended to increase local women’s participation in health promotion so that the improvement in maternal and child health can be achieved at a low cost even in remote places. People in remote communities have difficulty accessing healthcare due to Nepal’s high mountains and hills with poorly developed transportation and healthcare systems [4]. Therefore, local women are selected to work as volunteers in every village unit and are usually selected by their communities.

The volunteers initially receive 18 days of basic training and refresher training every five years on topics such as maternal and child health, and family planning [4]. The volunteers are also trained in specific topics if they are to provide specific healthcare services, e.g. administration of pregnancy test [10]. Although volunteers are often not compensated for their time, the duration of volunteering has increased from 1.7 to 3.1 hours per day between 2006 and 2014. Such increased working hours without matching incentives has led the volunteers to demand incentives, and yet the retention rate for volunteers is extremely high (97%). Over 52000 volunteers currently work across the country and are often the first point of contact for rural women, thereby filling a gap between the community and formal healthcare providers [16].

Volunteers continue to be a key resource for provision of maternal and child healthcare in rural communities of Nepal [17]. However, women from ethnic minority groups seem reluctant to use volunteers’ services, including local healthcare services [1, 3, 18]. For example, women from high-caste groups (Bramhin and Kshatri) are almost twice as likely to deliver at health facilities (48%) compared to low-caste groups (Dalits, 26%) [3]. Similar results have been observed for other ethnic minority groups such as Muslims and indigenous populations (Tamang, Chepang etc.) [3]. Literature review suggests women’s decision to use healthcare services is often shaped by a variety of factors: women’s autonomy and gender relationships [19, 20]; traditional health beliefs, including reliance on faith healers [12]; and skills and attitudes of healthcare providers [21]. However, there is a lack of empirical evidence about factors that lead to underuse of healthcare among ethnic minorities in rural Nepal [1, 3]. Understanding these barriers to accessing healthcare is important if healthcare services are to be suitable and acceptable for everyone in LMICs [20, 22].

To the best of our knowledge, this is the first study conducted in Nepal that aims to understand the factors that discourage the use of healthcare services by ethnic minority groups across the continuum of stakeholders from paid healthcare workers, volunteer healthcare providers and women service users. Employing qualitative methods to explore barriers to using maternal and child healthcare services, including sociocultural nuances that affect use of healthcare among ethnic minority groups in rural Nepal.

## Methods

We used qualitative methods, interviews and Focus Group Discussions (FGDs), to explore use of maternal and child healthcare services delivered by volunteers and the reasons for the underutilisation of these services. This section compares the study setting, outlines the ethical considerations posed by the study and explains how the data set was collected and analysed.

### Setting of the study

Two different communities from the hill and the Terai–the flatland region of Nepal bordering southern India—regions were selected. In the hill region, Dhading District was chosen, as the place is well known for its success in implementing the FCHV programme despite having a difficulty in terms of the distance to the nearest facility with some places located one to five hour walk away [23, 24]. In Dhading, Gajuri Village was chosen as a main study site with some additional interviews held with people from surrounding villages. Each village unit (the smallest unit of village) has one FCHV with a mix of ethnic groups: Bramhin, Dalit, Tamang and Chepang, which allowed us to capture diverse views.

In the Terai, Sarlahi District was chosen because the place has easy access to healthcare services compared to the hill region. The number of FCHVs in the Terai is dependent on its population, with one FCHV for about every 400 people. The district has a total population of 769, 729 with ethnic groups such as Tamang, Kshatri, Muslim and Madhesi [14]. Madhesi has its own caste division, which is beyond the scope of this paper. Yet inclusion of these diverse populations allows us to compare and contrast experiences of different ethnic communities regarding the use of healthcare services.

### Research ethics

Ethical approval was obtained from the Nepal Health Research Council on the 10^th^ May 2013 (registration number 32/2013). Informed consent was obtained from all participants. The purpose of the research was explained to each participant and each was provided with a ‘participant information sheet’ and a ‘written consent’ form. They were informed that their participation was voluntary. Written permission was sought from everyone. However, for the participants who could not read and write, the first author (SP) read out the informed consent form and obtained oral consent. The names and exact age of the participants have been removed from the manuscript. The individuals in this manuscript have given written informed consent (as outlined in PLOS consent form) to publish these case details, when possible.

### Data collection

Data were collected between April 2014 and September 2014 in two regions of Nepal using qualitative methods: interviews, FGDs and field notes. In total, 71 individuals participated in this study. Interviews were conducted with 20 FCHVs, 26 service users, and 11 health workers who had supervised the volunteers. An additional 15 FCHVs were included in four FGDs. Table 1 shows the number of participants in interviews and FGDs from the Hill and Terai regions.

**Table 1:** Participants involved in interviews and focus group discussions.

Study method	Type of study participants	Number of participants by location		Total
		Hill (Dhading)	Terai (Sarlahi)	
Interviews	FCHVs^+^	8	12	20
	Female service users	14	12	26
	Health workers (public)	4	3	7
	Health workers (private)	2	2	4
Focus Group Discussions	FCHVs	4*	11	15*
Total		32	40	71

+ FCHV- Female Community Health Volunteer

*1 person was interviewed and also attended a focus group.

A semi-structured topic guide was used to collect the data (Panday et al., 2017). Both Nepali and English versions of the topic guides are attached (S1 Appendix and S2 Appendix) and show questions used in the previous doctoral study [13], from which the current paper is developed. Most study participants were interviewed because they were based in remote areas, e.g. some volunteers were living a six-hours walk from the health centre (Table 2). All participants were purposely selected to reflect diversity in age, education and years of volunteer experience (Tables 2 and 3).

**Table 2:** Socio-demographic characteristics of Female Community Health Volunteers (FCHVs).

Respondents	Place	Type of Data	Age	Caste/ ethnicity	Education (in years)	Work Experience (years)	Walking distance to health centres
D1	Dhading	Interview	45–59	Brahmin	Literate	15	1hr
D2			≥60	"	"	"	2hrs
D3			45–59	"	"	"	20 min
D4			≥60	Tamang	0	"	5-6hrs
D5			45–59	Brahmin	2	16	1hr
D6			“	Chhetri	Literate	24	2 min
D7			30–44	Bhujel	“	"	30 min
D8			“	Brahmin	10	7	15 min by bus
S9	Sarlahi		“	Tamang	5	10	30–45 min
S10			“	"	0	"	1 hr
S11			“	"	"	19	"
S12			≥60	"	"	25	15 min
S13			45–59	Madhesi	"	19	10 min
S14			45–59	Gurung	Literate	"	25 min
S15			30–44	Chhetri	8	"	1 hr
S16			45–59	Brahmin	10	"	20 min
S17			≤30	"	9	3	1hr of cycling
S18			45–59	Lama	4	21	25 min
S19			“	Magar	0	19	1 hr
S20			“	Madhesi	10	26	15 min
FGD1	Dhading, Gajuri		≤30	Lama	"	4	1.5 hrs
			≤30	Brahmin	Intermediate	6	30 min
			45–59	Chepang	0	16	2 hrs
			“	Brahmin	Literate	15	1 hr
FGD2	Sarlahi, Harion		30–44	"	10	19	20 min
			45–59	"	0	"	10 min
			30–44	"	10	"	15 min
			45–59	"	8	"	30 min
			30–44	"	10	"	“
FGD3	Sarlahi- Lalbandi		≤30	"	12	3	20 min
			30–44	Gole	10	7	"
			45–59	Lama	"	2	30 min
			≤30	Brahmin	"	1	20min
			≤30	"	12	"	2 hours
			30–44	Chhetri	10	"	10 min

Literate- Able to read and write Nepali, < less than, ≤ less than or equal to, min- minute, hr-hour

**Table 3:** Demographic characteristics of service users (pregnant women or mothers).

Respondents	Place	Age	Ethnicity	Education	Age married	Working status	Distance from health centres (walk)	Number of children	Recent place of delivery
Woman1	Dhading	15–20	Dalit	2	16	Farmer	1 hour	0	Home
Woman2	“	>35	Tamang	0	22	Housewife	“	6	Home
Woman3	“	25–30	Chepang	0	14	“	“	3	Heath centre
Women4	“	“	"	0	13	Farmer	“	6	Home
Woman5	“	20–25	"	0	15	“	“	3	“
Woman6	“	15–20	Dalit	4	18	Housewife	45 min	Pregnant	-
Woman7	“	20–25	"	6	17	“	40 min	2	Heath centre
Woman8	“	“	"	0	20	Farmer	“	0	-
Woman9	“	30–35	Brahmin	12	20	Teacher	50 min	2	Home
Woman10	“	“	Tamang	0	15	Housewife	45 min	5	“
Woman11	“	25–30	Brahmin	6	14	Farmer	1 hour	4	Health centre
Woman12	“	20–25	"	12	16	Housewife	5 min	2	“
Woman13	“	“	"	10	19	“	10 min (bus)	1	“
Woman14	“	25–30	Dalit	12	21	“	15 min (bus)	2	“
Woman15	Sarlahi	15–20	Lama	7	17	“	30 min	1	Heath centre
Woman16	“	30–35	Muslim	0	13	“	1 hour	4	Home
Woman17	“	20–25	Brahmin	12	17	“	15 min	2	Heath centre
Woman18	“	“	Tamang	10	21	Farmer	5 min	1	“
Woman19	“	25–30	"	0	26	Housewife	30 min	2	“
Woman20	“	20–25	Brahmin	12	22	“	“	Pregnant	-
Woman21	“	25–30	Dalit	8	19	“	“	3	“
Woman22	“	20–25	Chhetri	12	19	“	“	1	“
Woman23	“	“	Newar	12	14	“	20 min	“	“
Woman24	“	“	Ale	12	23	“	20 min	Pregnant	-
Woman25	“	15–20	Madhise	0	14	“	30 min	2	Heath centre
Woman26	“	20–25	Magar	10	22	“	“	1	“

Table 2 shows FCHVs represented diverse ethnic groups with a majority being Brahmin (n = 17) followed by Tamang and other ethnic groups. There was no FCHV from the Dalit community.

Table 3 shows a majority of service users represented ethnic minority groups. In the hill villages, users were from Dalit, or indigenous communities such as Chepang, Tamang or Bhujel. In the Terai, they represented either the upper caste (Brahmin, Kshatri) or minority ethnic groups (Madhesi and Muslim), who were often illiterate. Service users were chosen if they were pregnant or mothers of children under two and living in the same area as the volunteers. Local health workers were also interviewed to reflect their views on volunteers’ services, and included individuals (6 males and 5 females) from both public health centres and Non-Governmental Organisations (NGOs) working in the study areas.

All the subjects (71 individuals) the first author approached participated in the study. While there were times when the subjects were a bit sceptical about the study, they agreed to participate as soon as the primary author explained to them about the research objectives and why she was undertaking the study. In total, only 15 women volunteers, who had to commute to participate in interviews, were paid each with NRs500 (US $3.46). The volunteers were reimbursed because the first author was able to secure a travel grant of US $1543 (£1180) from the University of Sheffield to cover the cost of local transportation for data collection within Nepal. Remaining interviews with health workers and service users including FGDs with volunteers were arranged at participants’ convenience and were not paid. They were held mostly at respondents’ homes but they were also undertaken in gardens, nearby fields or local teahouses. When possible, some snacks and refreshments (tea, biscuits and cold drinks) were arranged for the study participants.

All interviews and FGDs were conducted by SP in Nepali and ranged in duration from 15 minutes to about one hour. During the FGDs, a local assistant helped with note taking. A local assistant was hired in each study region to accompany the researcher and to assist her to locate study participants. Table 4 shows the demographic characteristics of health workers.

**Table 4:** Demographic characteristics of health workers.

HW	Place	Age	Position	Working institution	Work Experience (yrs.)	Caste/ethnicity
HW1	Dhading	44	Staff nurse	Government	13	Brahmin
HW2	“	"	ANM	“	22	“
HW3	“	56	AHW	“	35	“
HW4	“	54	DPHO	“	31	Muslim
HW5	“	28	ANM	NGO	6	Indigenous
HW6	“	33	Field Coordinator	“	8	“
HW7	Sarlahi	42	Senior AHW	Government	42	Madhesi
HW8	“	34	FCHV district supervisor	“	8	“
HW9	“	55	AHW	“	30	“
HW10	“	24	ANM	NGO	4	Indigenous
HW11	“	48	Field Coordinator	“	25	Brahmin

HW: Health Workers, PHC: Primary Health Care, SHP: Sub Health Post, ANM: Auxiliary Nurse Midwife, AHW: Auxiliary Health Worker, FCHV: Female Community Health Worker, DPHO: District Public Health Officer, CMA: Community Medicine Auxiliary, NGO: Non-Governmental Organisations, VDC: Village Development Committee.

Table 4 shows health workers represented a diverse ethnic group.

During the data collection, SP tried to ensure that her position, as an upper caste educated female from a foreign country, would not influence the participants’ complete disclosure of events, as social background is often reported to influence data collection and interpretations [25]. The researcher’s gender and nursing background might have given participants a feeling of being at ease, because both volunteers and service users openly discussed the reasons behind the underutilisation of healthcare services. In addition, her familiarity with the study environment and her previous research in the country might have helped her to approach the participants in a friendly manner, as all people approached to take part agreed to participate.

### Data analysis

The interviews were transcribed with help from local assistants. SP listened to the recordings and translated them. The data set was stored in NVivo10 for analysis. Data was analysed using thematic analysis [26, 27]. The data used in this paper is a part of the doctoral thesis that explored the role of volunteers in maternal health service provision in rural Nepal [13]. A key finding of that doctoral study was that the ethnic minority groups often did not use volunteers’ services despite their attempts to engage with them. Hence, this paper focuses on the perceived barriers to use healthcare by ethnic minority groups.

In order to increase the study’s rigor, multiple coding was applied to part of the data [28]. The third author coded a selection of the early transcripts in English independently and the codes were compared with those of SP and any discrepancies were discussed and resolved. In order to ensure internal validity, the data were triangulated across research methods (interviews and FGDs), respondents (FCHVs, their services users, and the local health workers) and study sites (hill/Terai). The convergence of themes across different groups of participants, places or methods was meant to enhance the validity of the research [29], but we also included divergent views. Our aim was to make the data analysis more comprehensive [25] thus providing a broader understanding of the use of FCHVs’ services. The study findings are illustrated with appropriate anonymous quotes using the following identifiers: HW (Health Worker), D (Dhading) and S (Sarlahi) each with the number of the participant.

## Results

The findings showing the perceived factors that discourage the use of healthcare services by ethnic minority groups are classified into five themes: a) lack of knowledge among service users; b) lack of trust in volunteers; c) traditional beliefs and healthcare practices; d) low decision-making power of women; and e) perceived indignities experienced when using health centres.

### a) Lack of knowledge among service users

While a majority of service users approached biomedical services (hereafter simply referred as healthcare services), many service users did not use such services despite their availability through volunteers or healthcare centres. Some volunteers were concerned that their service users particularly from indigenous ethnic minority groups such as Chepang and Tamang in the hill villages, and Madhesi and Muslims in Terai did not understand the importance of healthcare services and hence did not access them. Many Dalit and indigenous mothers (6 out of 14) in the hills delivered at home, compared to only one Muslim mother in the Terai (Table 3). This happened despite referral to health centre by the volunteers. Also a volunteer reported that some parents were reluctant to immunise their children:

*“I walked two to three hours up hill to provide polio drops. The woman did not accept the medicine. She said, ‘Our children do not need medicine, they will survive’. It took me three hours to reach them. I did not go there through my self-interest, yet I could not do anything about it.” FCHVD7*.

This was consistent with views of some service users for whom the concept of modern medicine was new and hence they did not see its value. A Tamang mother reported that she had not vaccinated her children:

*“People in the village say*, *“why do you need to vaccinate*? *People from the past are surviving without a single injection*.*” For this reason*, *my two kids are not vaccinated*.*” WomanD10*.

Another Tamang woman reported that she did not need any contraception, as she had learned from her previous pregnancies that she would not get pregnant for the next two years:

*“I won’t menstruate till the baby is two years. I menstruate exactly after 2 years then I conceive. It has always been like this. I have four kids and was the same for all. Now, I am on the 11 months.” WomanD10*.

Health workers also revealed a similar attitude toward ethnic minority groups. They reported difficulties in communicating and dealing with Chepang, who reportedly had substantially lower level of awareness of hygiene and healthcare compared to the general Nepali population. A health worker commented:

*“It is mainly associated with the level of awareness of healthcare services among people. In this ward number 4, hygiene is very poor among the Chepang community. The lack of enthusiasm for their work among the Chepang leads to low participation in health related activities by Chepang women.” HW5*.

### b) Lack of trust in the volunteers

Lack of trust on volunteers’ services was observed. This was related to volunteer’s inability to communicate health messages. Some people, including those reasonably well educated, strongly rejected the volunteers’ offers of medicine. This was reported in the Terai, where volunteers’ distribution of medicine to prevent filariasis infection from mosquito bites was seen as unnecessary or detrimental:

*“Some people ordered us, ‘you go away! you go away! you go away! we don’t want to take your tablets. You brought this medicine to kill us.’ The people, who reacted like this were the ones who were better educated. They didn’t take the medicine for filariasis.” FGD3 Participant4*.

One of the reasons for such negative reactions could be due to perceived unknown side effects of the filariasis medicine, which might or might not have been true.

*“Some children became unconscious, some had fever and some developed typhoid due to that medicine. So people came to us several times to complain about the matter.” VolunteerS9*.

Another reason could be due to the volunteers’ inability to communicate the importance of medicine as many volunteers were illiterate (Table 2) and had received only two weeks of training at the beginning of their volunteering. This is evident when some volunteers acknowledged that the current generation of women are more educated and confident than the FCHVs themselves, thus often not needing their help. A FCHV commented:

*“There are educated girls in the mothers’ group now* [a group of women gathered from a village to gain health information]. *I ask one of them to read and ask the rest of the group to listen to her and learn from that*. *We have become old and forgotten the training*. *…*. *If there is anything wrong*, *then they correct us*.*” FGD2Participant2*.

We also found that volunteers’ inability to communicate the health messages could additionally be related to the quality of training they receive. A health worker admitted that sometimes volunteers were not adequately prepared for their work, as they were simply trained and paid for a day instead of the actual training duration of three to four days:

*“The training duration for FCHVs is reduced contrary to the given guidelines, so as to save money from giving allowances to them. If there is training for three or four days then, it would be reduced to one day.”HW11*.

The above findings show that the use of health services among pregnant women and mothers is affected by their awareness of healthcare services as well as skills among health workers and volunteers in communicating the importance of healthcare services.

### c) Traditional beliefs and healthcare practices

In the hill villages, visiting a faith healer was a common practice among all ethnic groups. In addition, many Dalits in the hill villages instead of visiting health centres for their illnesses, they believed that praying to God would heal them. In the Terai communities, Muslim families believed that use of modern healthcare services would anger their God, so they did not use them.

Almost half of service users reported that they went to faith healers for treatment before they sought care from a health centre. A woman from Chepang community commented:

*“I usually go to a faith healer for treatment. If that does not work, then I go to a hospital for further treatment.” Woman3D*.

A Dalit woman reported how her baby recovered after her visit to a faith healer:

*“My baby was too hot. I took him to a faith healer for treatment. Then he was okay. I didn’t take him to a hospital.” WomanD1*.

While some service users believed in faith healers, some Dalits believed in the healing power of the Bible, as they had recently been converted to Christianity.

*“Some people told us that if we believe in the Bible, we don’t need to go to faith healers. What to do, I don’t know. They told us we need to have faith on Bible, then our children do not become ill. Or, even if they become ill, if we pray then it will heal.” As we don’t know how to read, they (Christians) read for us.” WomanD5*.

In one case, according to the mother from a Dalit community, family members kept praying for the life of the baby who was ill instead of taking him to hospital. Eventually, the baby died.

*“The baby gasped once. His body became blue. He abnormally dragged the clothes on his body that was kept for praying, and then he rolled his eyes upwards. Then they (relatives) said that it was not necessary to take him to a hospital. He died in my brother-in-laws house.” WomanD8*.

While the Dalit mother regretted that she did not take her baby to a hospital, she appeared to have accepted the untimely death of her son as his fate:

*“Even if we had him taken to a hospital he could not have reached to the Thati (close by public place) on time. His time had arrived. He might not have lived even if we had taken him to a hospital. However, he was not taken (to the hospital), and that was a mistake.” WomanD8*.

Similarly, in the Terai, the volunteers reported that service users from Muslim families were reluctant to accept immunisation, as they believed that immunisation would anger their god, and this belief was reinforced by mothers-in-laws.

*“Some service users say, ‘the wound from immunization makes our God angry. I don’t want to have any injections. Why do you ask me to have an injection? If my mother-in law or father-in-law knew about this, they would be annoyed.’” (FGD2 Participant5)*.

Another case was that of a pregnant Muslim woman with four children, all delivered at home without any assistance from health workers. She reported that her mother-in-law would move her pregnant abdomen to correct the baby’s position, unaware that such practice could be harmful:

*“I never felt a need to go to a health centre*. *The health workers might look at me and rotate my baby*. *That much can be done by my* mother-in-law *at home so I don’t go*.*” WomanS2*.

While the above evidence shows how traditional beliefs among service users and their family members discourage them from using healthcare services, the findings also demonstrate that how women have to rely on approval from their family members to access healthcare services. Below we present how family influences women’s use of healthcare services.

### d) Low decision-making power of women

Some women complained of their inability to access healthcare when they needed care. Husbands, mothers-in-laws or relatives frequently perceived healthcare services as irrelevant or unnecessary to them and their families. In addition, traditional beliefs such as sons are better than daughters had forced women to repeatedly become pregnant in order to give birth to a boy.

A volunteer reported how mothers-in-laws were reluctant to accept their advice to support their pregnant daughters:

*“The government tells us ‘new mothers should take rest for a month, do not wash clothes.’ We say the same thing in the village. However, some say, ‘this woman (FCHV) has come to us as an advocate to provoke our daughters-in-law so that they would not have to work.’” (FCHVD3)*.

Similarly, despite awareness about possible complications related to frequent pregnancies, some women kept having children. A pregnant woman, already a mother of four children, reported that she was having another child in order to fulfil her husband’s wishes:

*“I got pregnant because my husband wanted another son*. *I have only one son*, *and you can’t rely on one…I am thinking to take an injection after the delivery of this child (to prevent further pregnancies)*. *Let’s see what my husband wishes*.*” WomanS2*.

In one case, a husband agreed to abort a pregnancy but the mother-in-law resisted, who wanted an additional male child in the family.

*“My husband told me that I am very ill, so if I could not carry on with the pregnancy, then I could choose to abort. When I came home, I asked my mother-in-law, who said, ‘people have 6–7 sons, you have only one son, why do you want to abort? Your body is ill, if you throw the baby then you become more ill or you may die, so it is not necessary to do that.’” WomanD10*.

Despite the resistance from family members, volunteers encouraged women to use healthcare services, as illustrated by the flowing quote:

*“She reassured me whenever I had any difficulties. She is my relative and she used to repeatedly tell me about health visits and their importance.” WomanS15*.

After crossing the above hurdles, when some service users finally managed to present at health centres, they found that they had to face another obstacle, which is presented below.

### e) Perceived indignities experienced when using health centres

We found that negative experiences at health facilities among service users discouraged them from visiting health centres. Both volunteers and their service users reported how health workers’ rough attitude towards pregnant women and mothers dissuaded their use of healthcare services. Sometimes health workers were verbally abusive to users as they instructed them what to do and did not pay sufficient attention to their concerns. The following example demonstrates, how volunteers had to accompany mothers at health centres to ensure that they were at a low risk of being treated badly:

*“I go with pregnant women to hospital; while the staff scold the women I close their (women’s) mouths. Then I say to the health workers, ‘why do you scold them, these are poor women, they cannot go anywhere.”‘ FCHVD3*.

A mother of two children echoed this sentiment. She revealed that she had not taken her iron tablets as prescribed due to their bad taste, but she was scared that the health worker would shout at her if she revealed this. Therefore, she lied to a health worker:

*“I did not take those iron tablets. I didn’t like the taste. I had a stomach-ache, so I didn’t use them. I was worried that the health worker would scold me, so I told them that I had taken them. But, I threw them away.” WomanD7*.

However, health workers did not reveal anything negative regarding the volunteers or users’ experiences at health centres. The above examples show how the interaction of health workers, volunteers and service users shape the use of healthcare services by pregnant women.

## Discussion

To our knowledge, this is the first study, which has explored the role of volunteers to help poor people overcome barriers to the use of healthcare services in Nepal. The following key barriers to accessing maternal and child healthcare services by ethnic minority groups are discussed: a) lack of knowledge about health services among volunteers and service users; c) traditional beliefs and healthcare practices; d) low decision-making power of women; and e) perceived indignities experienced when using health centres. These are discussed below in comparison to studies from similar settings.

The first barrier to using healthcare was related to lack of knowledge about healthcare services in ethnic minority groups interviewed in this study. Their limited understandings of healthcare can to some extent be attributed to low levels of education of both the users and the volunteer healthcare providers (Tables 2 and 3). This is supported by national statistics regarding adult literacy rates for women (44.5%) or volunteers (42%) compared to men (71.6%) [14, 30]. It is likely that education limited volunteers’ ability to communicate the importance of healthcare messages to their users who had poor knowledge of maternal health and has been reported earlier [31]. Therefore, educational programmes targeted at ethnic minority users could enhance their understanding and use of healthcare services [32–34].

The volunteers in our study reported that some service users did not understand the purpose of the medicine they were distributing to prevent filariasis—a mosquito-borne disease that could cause painful and disfiguring swelling of the legs and genital organs, hence many locals refused to take the drugs. The drugs were anticipated to cause serious side effects, which led the community members to refuse it. A similar finding was reported in rural Latin America, where the community refused antimalarial drugs distributed by volunteers who were unfamiliar with the drugs [35]. On the contrary, trained volunteers successfully distributed vitamin A tablets in Nepal [36], drugs for shistosomiasis in Kenya [37] and achieved a high level (89%) of compliance with health services in Uganda [38]. Therefore, volunteers should be provided with thorough training and on-going refresher courses [10, 39]. Volunteers’ training should also include instruction about possible side effects of the drugs that the volunteers distribute. If volunteers recognise any signs and symptoms of such drug interaction, they should be able to refer the sick individual to the local health centre, which has the capacity to treat the person. Unfortunately, many local health centres in Nepal are not well equipped to deal with medical emergencies.

The second barrier to accessing healthcare services was related to traditional health beliefs or practices among users or their family members that perpetuated harmful traditional practices. A Muslim woman in our study reported practices of rotating a pregnant abdomen to correct the baby’s position, which could be harmful to both mothers and children. Similarly, indigenous groups such as Chepang and Tamang reported visiting faith healers to treat their illness. Such practices have also been reported in earlier studies of Nepal [40], India [5], or Afghanistan [41]. Often the users preferred to continue traditional healthcare practices, including visiting faith healers because they were immediately available in times of ill health. This was more common among older household members in conservative families such as Muslim and Dalits. While faith healing practices entail indigenous knowledge and need to be understood in-depth [42], such practices are linked with growing health inequalities [12] and can have unintended consequences or even death [43]. We noted a death of a new-born because family members did not take him to a hospital and instead kept praying for his life. In the hill village, many Dalits had become Christian and believed that praying to God would heal their illnesses. Therefore, community health programmes should involve religious leaders and traditional faith healers to ensure that they would understand the value of healthcare services and refer community members to visit nearby health centres.

The third barrier to use of healthcare services was related to low decision-making power of women within families or society as a whole. We found that often a mother-in-law may decide the woman’s use of healthcare, who often perceived healthcare services as irrelevant or unnecessary to them and their families. This was common among indigenous populations such as Chepang, Tamang in the hill village and Muslim in the Terai. For example, some Muslim families were reluctant to immunize children because mothers-in-law believed that immunisation would anger their god. Such beliefs dissuaded women from utilising healthcare and are consistent with earlier studies that showed a woman, particularly in rural and the Terai regions, is less likely to make decisions about her own health and have less autonomy compared to woman from urban areas [33, 44]. This is due to power and knowledge differences between women and their mothers-in-law or husbands [33, 44], and is linked to higher maternal deaths [45]. Additionally, some mothers-in-law such as Tamang in our study preferred male babies over female babies and expected women to produce multiple male children. Such preference of a male child over female is common among many ethnic groups in Nepal and has caused a wide gender gap with its gender inequality ranking - 145th out of 187 countries [46]. Therefore, community health programmes aimed to increase uptake of maternal and child healthcare services should involve both mothers-in-law and husbands to ensure that they would support women to seek healthcare when needed.

We observed such power differences during our interviews too. Often husbands or mothers-in-law would respond to the questions directed at mothers or pregnant women. In order to stop such interference, the first author informed and explained to them about the interview process and the importance of speaking to the particular women in private. Therefore, future research and healthcare interventions should ensure that husbands or mothers-in-law become an integral part of such programmes directed at improving healthcare use among women.

The final barrier to accessing healthcare by women was related to perceived indignity by service users when presenting at healthcare centres. While the relationship between service users and healthcare providers was recognised as key to users’ experiences of accessing services, insensitivity of healthcare providers can cause barriers to service access. We found that sometimes health workers were verbally abusive to users as they instructed them what to do and did not pay enough attention to users’ concerns. Such behaviour could undermine personal dignity and discourage women from using healthcare services as seen in earlier studies [21, 22, 47]. Therefore, it is imperative that professional health workers are aware of the rights of communities and provide respectful care to vulnerable groups [21].

The findings should be interpreted cautiously as our study participants represented two small communities of Nepal and therefore may not represent the views of wider ethnic minority groups. Moreover, this was a challenging area to explore at one single point of time through one-off interviews or FGDs. We noted several unique social, cultural, and religious aspects of our study regions in which healthcare volunteers and those using their services live. The on-going religious practices and acceptance of faith healers within ethnic minority populations have implications in healthcare service use. Muslim, Dalit and indigenous women lacked knowledge around healthcare services. Religious practices and family members, especially mothers-in-law, were against the use of healthcare and were reported to discourage women from visiting health centers. Both volunteers and health workers in the study villages lacked appropriate knowledge and communication skills to deliver healthcare services. These findings remind us of the importance of identifying specific local challenges and opportunities to promote healthcare use among ethnic minorities. More prolonged work is necessary to study the relationship between ethnic minority users, and volunteers and paid healthcare workers using participatory and ethnographic approaches [8, 48]. Future research should also focus on distinctive issues related to ethnicity, gender and older adult women’s acceptance of healthcare services as they affect the use of maternal and child healthcare services.

Furthermore, little is known about the actual healthcare system in Nepal beyond “cultural influences.” While volunteers’ use as healthcare providers is predominantly based on a chronic shortage of healthcare workers, management of these volunteers in new Nepal is largely unknown. Nepal, now a Federal Democratic Republic country, has started a decentralisation process, but it is largely unknown how the decentralisation is going to affect the healthcare systems at the central, provincial and local levels of government. Urgent in-depth studies are needed if we want to meet the goal of universal healthcare coverage.

We believe that our study maintained its quality and rigour through the consideration of reflexivity, and inclusion of a broad sampling method combined with applications of triangulations (study design: interviews and focus groups; study areas: the hill and Terai; and study population: volunteers, service users and paid health workers) [28, 49]. This study not only identified key barriers that are relevant to ethnic minority groups but also barriers that can be shared with the other groups, such as women’s position in households, which is shared by many women in South Asia and around the globe.

## Conclusions

Access to healthcare services for ethnic minority groups who are vulnerable to health risks is an important issue for policy makers and international donor agencies. Our findings suggest a need to develop a common understanding among service users, local healthcare workers, and volunteers if we want to improve health service use among ethnic minority groups. As the role of CHWs such as FCHVs continue to be crucial in order to achieve universal healthcare coverage for maternal and child health in LMICs [9, 10, 50], FCHVs should be educated and equipped with effective communication skills. There is a need to create awareness about the roles of FCHV and their services among community people. Local health workers should be trained to deliver respectful maternity care. Service users, including traditional healthcare providers and family members (mother-in-law and husband) should be the target of health programmes that aim to raise awareness on the importance of seeking medical attention during pregnancy and childbirth, if we want to increase access to healthcare for the ethnic minority population and support Nepal in its attempts to address health inequalities. The findings also have relevance to similar resource poor settings.

## Supporting information

S1 Appendix A Nepali version of the topic guide that shows questions used in the previous doctoral study, from which the current paper is developed. (PDF)

S2 Appendix An English version of the topic guide used in the previous doctoral study, from which the current paper is developed. (PDF)

## Abbreviations

FCHV: Female Community Health Volunteer
LMIC: Low- & -Middle Income country
PHC: Primary Health Care
FGD: Focus group Discussion
WHO: World Health Organisation

## References

[1] Bennett L, Dilli RD, Pav G. Caste, Ethnic and Regional Identity in Nepal: Further Analysis of the 2006 Nepal Demographic and Health Survey. Calverton, Maryland, USA: Macro International Inc.; 2008.

[2] Bhandari P, Chan L. Socio-cultural Inequality in Women's Health Service Utilization in Nepal. Asia-Pacific Population Journal. 2016;31(2).

[3] Pandey JP, Dhakal M, S.K P, MS P. Maternal and child health in Nepal: the effects of caste, ethnicity, and regional identity: further analysis of the 2011 Nepal demographic and health survey: Ministry of Health and Population; 2013.

[4] Family Health Division. National female community health volunteer program strategy. Kathmandu: Family Health Division. Government of Nepal, 2010.

[5] Khan M, Hazra A, Bhatnagar I. Impact of Janani Suraksha Yojana on selected family health behaviors in rural Uttar Pradesh. Journal of Family Welfare. 2010;56:9–22.

[6] Chowdhury AMR, Bhuiya A, Chowdhury ME, Rasheed S, Hussain Z, Chen LC. The Bangladesh paradox: Exceptional health achievement despite economic poverty. The Lancet. 2013;382(9906):1734–45. 10.1016/S0140-6736(13)62148-0. . 24268002

[7] Najafizada SA, Labonte R, Bourgeault IL. Community health workers of Afghanistan: a qualitative study of a national program. Conflict & Health. 2014;8(26). 10.1186/1752-1505-8-26 25904976 PMC4405840

[8] Maes K, Closser S, Kalofonos I. Listening to Community Health Workers: How Ethnographic Research Can Inform Positive Relationships Among Community Health Workers, Health Institutions, and Communities. American Journal of Public Health. 2014;104(5):e5–e9. 10.2105/AJPH.2014.301907 . 24625167 PMC3987580

[9] Engel J, Glennie J, Adhikari SR, Bhattarai SW, Prasai DP, Fiona S. Nepal's Story Understanding improvements in maternal health London: Overseas Development Institute, 2013.

[10] Panday S, Bissell P, van Teijlingen E, Simkhada P. The contribution of female community health volunteers (FCHVs) to maternity care in Nepal: a qualitative study. BMC Health Services Research. 2017;17(1):623 10.1186/s12913-017-2567-7 28870185 PMC5584032

[11] Pradhan Y, Upreti SR, Pratap KC N, Kc A, Khadka N, Syed U, et al Newborn survival in Nepal: a decade of change and future implications. Health policy and planning. 2012;27(suppl_3):iii57–iii71. 10.1093/heapol/czs052. 22692416

[12] Jha N, Bhattarai S, Niraula S. Socio-cultural factors associated with morbidity and mortality: A study from Eastern Nepal. Health Renaissance. 2013;13(3):114–28.

[13] Panday S. The role of female community health volunteers in maternal health service provision in Nepal: a qualitative study. Sheffield: The University of Sheffield; 2016.

[14] CBS. National population and housing census 2011 (National Report). In: Secretariat NPC, editor. Kathmandu: Central Bureau of Statistics; 2012 p. 1–4.

[15] Gurung H. The Dalit Context. Occasional Papers in Sociology and Anthropology. 2005;9:1–21. 10.3126/opsa.v9i0.1133

[16] Government of Nepal, Ministry of Health & Population. Female Community Health Volunteers Nattional Survey Report In: (DoHS) DoHS, editor. Teku, Kathmandu, Nepal: Family Health Division; 2014 p. 1.

[17] Government of Nepal, Ministry of Health & Population. Female Community Health Volunteers Nattional Survey Report In: (DoHS) DoHS, editor. Teku, Kathmandu, Nepal: Family Health Division; 2014 p. xvi.

[18] Yuan B, Målqvist M, Trygg N, Qian X, Ng N, Thomsen S. What interventions are effective on reducing inequalities in maternal and child health in low- and middle-income settings? A systematic review. BMC Public Health. 2014;14(1):634 10.1186/1471-2458-14-634 24952656 PMC4083351

[19] Higginbottom GMA, Mathers N, Marsh P, Kirkham M, Owen J, Serrant-Green L. Young people of minority ethnic origin in England and early parenthood: views from young parents and service providers. Social science & medicine. 2006;63(4):858–70. 10.1016/j.socscimed.2006.03.011. 16678322

[20] Say L, Raine R. A systematic review of inequalities in the use of maternal health care in developing countries: examining the scale of the problem and the importance of context. Bulletin of the World Health Organization. 2007;85:812–9. 10.2471/BLT.06.035659 18038064 PMC2636485

[21] Devkota HR, Murray E, Kett M, Groce N. Are maternal healthcare services accessible to vulnerable group? A study among women with disabilities in rural Nepal. PLOS ONE. 2018;13(7):e0200370 10.1371/journal.pone.0200370 30005080 PMC6044538

[22] Miteniece E, Pavlova M, Rechel B, Groot W. Barriers to accessing adequate maternal care in Central and Eastern European countries: a systematic literature review. Social Science & Medicine. 2017;177:1–8. 10.1016/j.socscimed.2017.01.049. 28152420

[23] District Health Office. District Profile. Dhading: Department of Health Services, Mid-Regional Directorate, 2014.

[24] Department of Health Services. Annual Report 2013/2014. Teku, Kathmandu: Department of Health Services, Ministry of Health and Population, Government of Nepal, 2014.

[25] Mays N, Pope C. Qualitative research in health care: Assessing quality in qualitative research. BMJ: British Medical Journal. 2000;320(7226):50 10.1136/bmj.320.7226.50 10617534 PMC1117321

[26] Braun V, Clarke V. Using thematic analysis in psychology. Qualitative Research in Psychology. 2006;3(2):77–101. 10.1191/1478088706qp063oa

[27] Ritchie J, Lewis J. Qualitative research practice: A guide for social science students and researchers: Sage Publications Limited; 2003.

[28] Barbour RS. Checklists for improving rigour in qualitative research: a case of the tail wagging the dog? BMJ. 2001;322(7294):1115–7. 10.1136/bmj.322.7294.1115 11337448 PMC1120242

[29] Lambert SD, Loiselle CG. Combining individual interviews and focus groups to enhance data richness. Journal of Advanced Nursing. 2008;62(2):228–37. 10.1111/j.1365-2648.2007.04559.x 18394035

[30] New ERA, USAID, Government of Nepal. An analytical report on national survey of female community health volunteers of Nepal. New ERA, 2007.

[31] Deo KK, Paudel YR, Khatri RB, Bhaskar RK, Paudel R, Mehata S, et al Barriers to utilization of antenatal care services in Eastern Nepal. Frontiers in public health. 2015;3:197 10.3389/fpubh.2015.00197 26322302 PMC4536371

[32] Choulagai B, Onta S, Subedi N, Mehata S, Bhandari GP, Poudyal A, et al Barriers to using skilled birth attendants' services in mid-and far-western Nepal: a cross-sectional study. Bmc International Health and Human Rights. 2013;13(1):49 10.1186/1472-698X-13-49. 24365039 PMC3878020

[33] Furuta M, Salway S. Women's position within the household as a determinant of maternal health care use in Nepal. International Family Planning Perspectives. 2006;32(1):17–27. 10.1363/ifpp.32.017.06 WOS:000237148000003. 16723298

[34] Hussein J, Lovney K, Margaret A, Stephen M. What kinds of policy and programme interventions contribute to reductions in maternal mortality? The effectiveness of primary level referral systems for emergency maternity care in developing countries. EPPI-Centre: [Department for international development](DFID), 2011 June 2011. Report No.

[35] Ruebush II TK, Weller SC, Klein RE. Qualities of an ideal volunteer community malaria worker: a comparison of the opinions of community residents and national malaria service staff. Social Science & Medicine. 1994;39(1):123–31. 10.1016/0277-9536(94)90172-4. 8066483

[36] Curtale F, Siwakoti B, Lagrosa C, LaRaja M, Guerra R. Improving skills and utilization of Community Health Volunteers in Nepal. Social Science & Medicine. 1995;40(8):1117–25. 10.1016/0277-9536(94)00172-P. 7597465

[37] Odhiambo GO, Musuva RM, Odiere MR, Mwinzi PN. Experiences and perspectives of community health workers from implementing treatment for schistosomiasis using the community directed intervention strategy in an informal settlement in Kisumu City, western Kenya. BMC Public Health. 2016;16(1):986 10.1186/s12889-016-3662-0 27634152 PMC5025566

[38] Chami GF, Kontoleon AA, Bulte E, Fenwick A, Kabatereine NB, Tukahebwa EM, et al Community-directed mass drug administration is undermined by status seeking in friendship networks and inadequate trust in health advice networks. Social Science & Medicine. 2017;183:37–47. 10.1016/j.socscimed.2017.04.009. 28458073 PMC5446315

[39] MoHP. National health policy 2071. Ramshahpath, Kathmandu: Ministry of Health and Population; 2015.

[40] Khatri RB, Dangi TP, Gautam R, Shrestha KN, Homer CS. Barriers to utilization of childbirth services of a rural birthing center in Nepal: a qualitative study. PLoS One. 2017;12(5):e0177602 10.1371/journal.pone.0177602 28493987 PMC5426683

[41] Trani J-F, Bakhshi P, Noor AA, Lopez D, Mashkoor A. Poverty, vulnerability, and provision of healthcare in Afghanistan. Social Science & Medicine. 2010;70(11):1745–55. 10.1016/j.socscimed.2010.02.007. 20359809

[42] Subedi M. Indigenous knowledge and health development in Nepal 1. State, Society and Health in Nepal: Routledge India; 2018 p. 61–74.

[43] Wasti H, Kanchan T, Acharya J. Faith healers, myths and deaths. Medico-Legal Journal. 2015;83(3):136–8. 10.1177/0025817215580908 25872779

[44] Acharya D, Bell J, Simkhada P, van Teijlingen E, Regmi P. Women's autonomy in household decision-making: a demographic study in Nepal. Reproductive Health. 2010;7(1):15 10.1186/1742-4755-7-15 20630107 PMC2914657

[45] Lowe M, Chen D-R, Huang S-L. Social and Cultural Factors Affecting Maternal Health in Rural Gambia: An Exploratory Qualitative Study. PLOS ONE. 2016;11(9):e0163653 10.1371/journal.pone.0163653 27661617 PMC5035064

[46] United Nations Development Programme (UNDP). Human Development Reports. Measuring inequality: Genderrelated Development Index (GDI) and Gender Empowerment Measure (GEM) 2013 [cited 2013 28 August]. Available from: http://hdr.undp.org/en/statistics/indices/gdi_gem/.

[47] Oosterveld‐Vlug MG, Pasman HRW, van Gennip IE, Muller MT, Willems DL, Onwuteaka‐Philipsen BD. Dignity and the factors that influence it according to nursing home residents: a qualitative interview study. Journal of Advanced Nursing. 2014;70(1):97–106. 10.1111/jan.12171 23711199

[48] Maes KC, Kohrt BA, Closser S. Culture, status and context in community health worker pay: Pitfalls and opportunities for policy research. A commentary on Glenton et al(2010). Social Science & Medicine. 2010;71(8):1375–8. 10.1016/j.socscimed.2010.06.020 20667639 PMC6211553

[49] Mays N, Pope C. Rigour and qualitative research. BMJ: British Medical Journal. 1995;311(6997):109 10.1136/bmj.311.6997.109 7613363 PMC2550154

[50] WHO. Using lay health workers to improve access to key maternal and newborn health interventions in sexual and reproductive health. 2013.

