# Supplementary material for: Perceived barriers to accessing Female Community Health Volunteers’ (FCHV) services among ethnic minority women in Nepal: A qualitative study
Source: PLoS One. 2019 Jun 10;14(6):e0217070. doi: 10.1371/journal.pone.0217070 (PMC6557479; doi:10.1371/journal.pone.0217070)
Supplement: S1 Appendix — (PDF) [file pone.0217070.s001.pdf]

सहभागी जानकारी पत्र

नेपालको मातृ स्वास्थ्य सुधारमा महिला स्वास्थ्य स्वयं सेविकाको (म.स्वा.से) भूमिका

तपाइहरुलाई यस अनुसन्धानमा भाग लिन अनुरोध गरिएको छ । यस अनुसन्धानमा भाग लिनु पूर्व यो अनुसन्धान किन गरिदैछ र यसमा के गरिन्छ भन्ने बारेमा जान्नु जरुरी छ। तलका जानकारीहरु राम्रोसँग पढेर (सुनेर) यसका बारेमा प्रष्ट नभएमा वा थप जिज्ञासाका लागि निर्धकतापूर्वक मलाई सोध्न सक्नु हुनेछ। तपाइ यस अनुसन्धानमा भाग लिने अथवा नलिने भन्ने निर्णय गर्ने पूर्ण अधिकार राख्नुहुन्छ।

अनुसन्धानको उद्देश्य

यो अनुसन्धान नेपालमा मातृ स्वास्थ्य सुधारमा महिला स्वयं सेविकाको (म.स्वा.से) भूमिका लगायत म.स्वा.से. मार्फत सेवा लिने आमाहरुको साथै स्वास्थ्य कर्मीको प्रतिकृया बुझ्नको लागि गरिएको हो । समुदायमा मातृ स्वास्थ्य सेवा प्रदान गर्ने म.स्वा.से. लगायत सरोकारवालाहरुको विचार र अनुभवको साथै उनिहरुको सम्बन्ध बुझ्ने प्रयत्न गरिएको छ।

यो अन्तर्वाता पूरा हुन ४५-६० मिनेट लाग्नेछ तर तपाइलाई थप समय चाहिएमा लिन सक्नु हुनेछ।

किन मलाई छानिएको ?

तपाईलाई यस अन्तर्वातामा भाग लिन अनुरोध गर्नुको कारण तपाई मातृ स्वास्थ्य सेवा प्रदान गर्दै हुनुहुन्छ । तपाइ जस्तै अरु व्यक्तिहरु समक्ष पनि यि प्रश्न राखिने छन।

के मैले भाग लिन जरुरी छ ?

यो अनुसन्धानमा भाग लिने अथवा नलिने भन्ने निर्णय तपाईमा निहित छ । यदि तपाईले यस अनुसन्धानमा सहभागी हुने निर्णय गर्नु भएमा तपाइलाई यो जानकारी पत्र दिएर केहि प्रश्नहरु सोधिने छन्।

मैले यस अनुसन्धानमा भाग लिए भने के हुन्छ ?

तपाइको कार्य अनुभवका बारेमा केही जानकारी लिनेछ। तपाइको यो अन्तर्वार्ता रेकर्ड गरिनेछ।

कसरी रेकर्ड मिडीयाको प्रयोग गरिनेछ ?

तपाईको अडियो रेकर्डलाई यस अनुसन्धानको प्रयोजनका लागि मात्र प्रयोग गरिनेछ। जस्तै, कक्षा र सम्मेलन, अन्य अवस्थामा यिनको प्रयोग गरिने छैन। यो अनुसन्धान बाहिरका मान्छेलाई यो रेकर्डिङ उपलब्ध हुने छैन। यस रेकडिङको केही भाग अनुसन्धानका कममा जस्ताको तस्तै राख्न सकिनेछ तर त्यसबाट तपाइहरुको नाम अथवा चिनारी हटाइने छ।

यस अनुसन्धानमा भाग लिनुका संभावित बेफाइदा

साधारणत यस अनुसन्धानमा भाग लिनुको कुनै बेफाइदा छैन तर तपाइको कार्य अवधिमा नराम्रो अनुभव भए त्यसको सम्झनाले तपाइलाई केही नराम्रो अनुभव दिन सक्ने छ।

यस अनुसन्धानमा भाग लिनुको फाइदाहरु
जब कि यस अनुसन्धानमा भाग लिनुको कुनै प्रत्यक्ष फाइदा छैन, यो आशा गरिन्छ कि यस खोजले समुदायमा म.स्वा.से प्रदान गर्दा त्यसलाई सहज बनाउने तत्वहरु साथै त्यसमै परी आउने अप्ठ्याराहरुका साथै उनीहरुको कार्य अनुभव र उनीहरुलाई सो कार्य गर्न प्रेरित गर्ने तत्वहरुको बारेमा जानकारी हासिल गर्न सजिलो हुनेछ । यसले नीति निर्मातालाई समुदायमा मातृ स्वास्थ्य सेवा कार्यक्रम सुधार गर्न सहज बनाउने छ ।

केही जिज्ञासा भएमा
केही जिज्ञासा भएमा तपाईहरुले मेरो फोन नं ९८४१५२८५०५ (सरिता पाण्डे) मा सम्पर्क गर्नु होला । यदि मेरो प्रतिक्रिया तपाइहरुलाई चित्त नबुझेमा मेरा सुपरिवेक्षक प्राध्यापक पदम सिम्खडा अथवा नेपाल स्वास्थ्य अनुसन्धान परिषदमा सम्पर्क गर्न सक्नु हुनेछ ।

अन्त्यमा,

यो अनुसन्धानमा आएका प्रतिक्रियाहरु पूर्ण गोपनियता राखिने छ ।

यो अनसन्धान २०७२ सालको पुष मसान्त भित्र सकिने अनुमान छ । जसको एउटा प्रतिलिपी नेपाल स्वास्थ्य अनुसन्धान परिषद र सम्बन्धित स्वास्थ्य संस्थामा बुझाउने छु ।

यो अनुसन्धानलाई नेपाल स्वास्थ्य अनुसन्धान परिषदबाट अनुमति प्राप्त छ । सेफिल्ड विश्वविद्यालयले यस अनुमतिलाई मान्यता दिने भएकाले पूनः अनुमोदनको जरुरी हुदैन ।

थप जानकारीका लागि
सरिता पाण्डे, कुपण्डोल, ललितपुर (+९७७-९८४१५२८५०५) वरिष्ठ प्राध्यापक पदम सिम्खडा, (सेफिल्ड विश्वविद्यालय +४४१०१ ११४२२२०७५२)

यो जानकारी पत्र पढ्न (सुन्न) समय दिनु भएकोमा धन्यवाद ।

सहभागी अनुमति पत्र

अनुसन्धानको शिर्षक

नेपालको मातृ स्वास्थ्य सुधारमा महिला स्वास्थ्य स्वयं सेविकाको (म.स्वा.से) भूमिका

अनुसन्धान कर्ताको नाम

सरिता पाण्डे

सुपरिवेक्षक

पदम सिम्खडा, इडविन भान तेजलिङ्गेन

सहभागी चिनारी पत्र

१. मैले यो अनुसन्धान सम्बन्धि **सहभागी जानकारी पत्र** पढे र यस सम्बन्धि प्रश्नहरु राखे । ☐

२. मेरो सहभागीता स्वेच्छिक हो र म कुनै पनि वेला यो अनुसन्धानमा भाग लिनबाट पछि हट्न सक्छु । ☐

३. मेरा विचारहरु अत्यन्तै गोपनिय राखिनेछ भन्ने मैले बुझेकि छु । मेरो व्यक्तिगत चिनारी बिना मेरो विचारहरु यस अनुसन्धान कर्ताहरुले प्रयोग गर्न पाउने छन् । मेरो नाम यस अनुसन्धानसंग जोडिने छैन । ☐

४. मेरा विचारहरु भविष्यमा यस अनुसन्धान सम्बन्धि अध्ययन तथा प्रकाशनमा प्रयोग गर्न म पूर्ण सहमत छु । ☐

५. म यो अनुसन्धानमा सहभागी हुन पूर्ण सहमत छु । ☐

सहभागीको नाम　　　　मिती　　　　हस्ताक्षर

अनुसन्धान कर्ताको नाम　　　　मिती　　　　हस्ताक्षर

सरिता पाण्डे

जानकारी संकलन (नीति निर्माता)

अनुसन्धानका सामान्य प्रश्नहरु

कसरी म.स्वा.स्व.से. ग्रामिण नेपालमा कार्य गर्दैछन् ?

मातृ स्वा.सेवा प्रदानमा म.स्वा.से. को भूमिकामा उनका सुपरिवेक्षक साथै सेवा ग्राहिहरुको के-कस्तो अनुभव र विचार छ ?

उद्देश्य

१. ग्रामिण नेपालमा मातृ स्वास्थ्य सेवा प्रदानमा म.स्वा.से. गतिविधिहरु पहिचान गर्ने

२. ग्रामिण नेपालमा मातृ स्वास्थ्य सेवा प्रदान गर्ने विशेष गरी तिनीहरुले बेहोर्नु पर्ने समस्या र तिनका कार्यलाई सहज बनाउने तत्वहरुको जानकारी लिने

३. म.स्वा.से. लाई कार्य गर्न प्रेरणा दिने तत्वहरु

४. मातृ स्वास्थ्य सेवा प्राप्त गर्दा महिलाहरुको म.स्वा.से. प्रतिको धारणा

जानकारी संकलनका लागि आवश्यक चेकलीष्ट (नीति निर्माता)

उमेर

जात/वर्ग

धर्म

शिक्षा

कार्य अनुभव (वर्षमा)

१) म.स्वा.से. नेपालमा तपाईको विचारमा मा.स्वा. सुधार हुनमा म.स्वा.से. को के-कस्तो भूमिका छ ?

२) म.स्वा.से. कार्यक्रमको सबल (बलियो) पक्ष र कमजोर पक्ष के-के हुन ?

३) म.स्वा.से. कार्यक्रमलाई प्रेरक (प्रेरणा दिने) बनाउने तत्वहरु के-के हुन् ?

४) धेरै जसो म.स्वा.से. पद नत्यागनु अथवा कामलाई निरन्तरता दिनुको कारण के हुन् ?

५) तपाइको विचारमा म.स्वा.से. कार्यक्रम कस्तो हुनुपर्छ र किन ?

६) तपाईले म.स्वा.से. कार्यक्रमको भविष्य कस्तो देख्नुहुन्छ र किन ?

७) यो कार्यक्रम चलाउन महिला नै हुनुपर्छ , किन ?

जानकारी संकलनका लागि आवश्यक चेकलीष्ट (महिला स्वास्थ्य स्वयं सेविका)

उमेर

जात/वर्ग

सामाजिक/आर्थिक/स्थिती

धर्म

शिक्षा

कार्य अनुभव (वर्षमा)

भाषा

सिप/तालिम

पारिश्रमीक

औसत दुरि सेवा प्रदानका लागि

सेवा ग्राहिसंग प्रयोग गर्ने भाषा

बच्चाको संख्या / (birth place)

घर / (family type)

शौचालयको व्यवस्था

महिला स्वास्थ्य स्वयं सेविका (म.स्वा.से.) सँगको अर्न्तवार्ता

१. म.स्वा.से. भन्नाले के बुभ्नु हुन्छ ? म.स्वा.से. हुँदा आफ्नो भूमिकाको बारेमा के सोच्नु भएको छ ?

२. मलाई म.स्वा.से. हुँदाको तपाईको अनुभव बताइदिनुस न । म.स्वा.से. भएर काम गर्दा तपाइले अनुभव गर्नुभएको तिता-मीठा दुवै कुराहरु जान्न चाहन्छु ।

३. म.स्वा.से. भएर काम गर्दा तपाइको कुनै एक दिनको कार्य शैली बताइदिनुसन । तपाइले प्राय जसो के काम गर्नुहुन्छ ?

४. मातृ स्वास्थ्य सम्वन्धि कुनकुन सेवा प्रदान गर्नुहुन्छ ?

५. हप्तामा कति घण्टा जति सेविका भएर बिताउनु हुन्छ ?

६. तपाई स्वयं सेविका बन्नु भन्दा पहिले यस गाउँमा (गर्भवति) आमाहरुको अवस्था कस्तो थियो ? तपाइ स्वयं सेविका भए पछि के भिन्नता अनुभव गर्नुभएको छ?

७. महिलाहरुलाई गर्भवती जाँच गराउन जाने बनाउनका लागि तपाइहरुले के-के गर्नु भएको छ ?

८. महिलाहरुले आमा बन्ने समयमा तपाइहरुको के भूमिका छ ? बच्चा पाउने बेलामा तपाइहरुले के कस्तो सेवा दिनुहुन्छ ?

९. भर्खर बच्चा जन्मेको आमासँग भेट भएमा के भन्नु हुन्छ ?

१०. तपाइ गाउँका महिलाहरु समक्ष कसरी पुग्न सक्नु भएको छ ? यदि छैन भने किन ? किन गाउँका सदस्य अथवा महिलाहरुले तपाइको सेवा रुचाउँछन, मन पराउँछन् ? (पहुँच, संचार,.......) तपाई गाउँका महिलाहरुसँग कसरी सम्बन्ध बनाउनु हुन्छ ? (आमा समूह, अन्य संस्थासँगको सहकार्य)

११. यदि महिलाहरु सेवा लिन आउँदैनन् अथवा तपाइलाई रुचाउँदैनन् भने किन ?

१२ . तपाई महिला भएकोले वा तपाइको जात, सामाजिक अवस्था, धर्मको कारणले तपाइको काममा कुनै प्रभाव पारेको छ ?

१३. तपाइले सधै गर्ने कार्यमा कुनै थप काम थपिएको छ ? नयाँ कामको बारेमा तपाइको के विचार छ ?

१४. तपाइसग तपाइको दैनिक काममा सहयोग पुऱ्याउने चिजहरु के के छन्? (जस्तै औषधी अन्य वस्तुहरु) यी चिजहरु कसले उपलब्ध गराइरहेछ ? खासमा स्वास्थ्य संस्थाले दिनु पर्ने तर कहि कहि गा.वि.स. तथा निजी संस्था छन्

१५. तपाइको काममा स्थानिय संस्था र गा.वि.स.ले के-कस्तो सहयोग गरेका छन् ?

१६. कहिले र कतिको तपाइको सुपरिवेक्षकहरु तपाइको कामको सुपरिवेक्षण गर्न आउँछन् ?

१७. कस्ता-कस्ता (कुन कुन) कामलाई सुपरिवेक्षण गरिन्छ ?

१८. तपाइले काममा के कस्ता अप्ठ्यारा परिस्थितीको सामना गर्नु भएको छ ?

१९. तपाइलाई कुनै समस्या पर्दा भनसुन गर्न कहाँ जानुहुन्छ ?

२०. के महिला स्वा.स्व.से. महिला नै बन्नु पर्छ ? किन त्यस्तो लाग्छ ?

२१. तपाइ किन स्वयं सेविका बन्नु भयो ?

२२. तपाइ कसरी म.स्वा.से. बन्नु भयो ? के त्यहाँ महिला समूह/आमा समूह थियो ? के त्यो छनौट प्रक्रिया सही थियो ? अहिले कस्तो व्यवस्था छ ? number of days of training, types of training

२३. के ले तपाइलाई आमा र बच्चाहरुको सेवा गर्नलाई प्रेरणा दियो ?

२४. तपाइ किन यो सेवा दिन उत्साहित हुनुहुन्छ ?

२५. मातृ स्वास्थ्य सुधार गर्ने उद्धेश्यले गाउँमा राम्रो सेवा दिनका लागि महिला स्वा.से. लाई सक्षम बनाउन के कुराको आवश्यकता देख्नु भएको छ ? (के भैदियो भने तपाइहरुले सजिलै राम्रोसँग काम गर्न सक्नुहुन्छ ?)

महिलासंगको अन्तर्वाता

जानकारी संकलनका लागि आवश्यक चेकलीष्ट

उमेर
जात/वर्ग
सामाजिक/आर्थिक/स्थिती
धर्म
शिक्षा

काम

भाषा

बच्चाको संख्या / (birth place)

घर / (family type)

शौचालयको व्यवस्था

तपाइ आफूलाई सन्चो नहुदा कहाँ जानुहुन्छ र किन ?

तपाइहरुले कसरी मातृ स्वास्थ्य सेवा प्राप्त गर्नु हुन्छ ? के तपाइले म.स्वा.से.बाट सेवा प्राप्त गर्नु भएको छ ?

तपाइले म.स्वा.से. बाट कुनकुन सेवा प्राप्त गर्नु भएको छ ? किन जानु हुन्छ म.स्वा.से. कहाँ

कसरी यी स्वयं सेविकाहरुले सहयोग गर्छन् ।

कस्तो-कस्तो किसिमको सेवा तपाइहरुले म.स्वा.से. बाट प्राप्त गर्नुहुन्छ ?

के म.स्वा.से. तपाइहरु भएको ठाउँमा आउँछन् ? अन्तिम पटक के का लागि म.स्वा.से. लाई भेट्नु भयो ?

तपाइ लाई म.स्वा.से. को काम कस्तो लाग्छ ?

के कुराले तपाइलाई म.स्वा.से. को विचार महत्वपूर्ण लाग्छ ?

तपाइलाई कुन सेवा महत्वपूर्ण लाग्छ र किन ? यदि तपाइलाई कुनै सेवा मन पर्दैन भने किन ?

सहभागी अनुमति पत्र (सामूहिक छलफल)

अनुसन्धानको शिर्षक

नेपालको मातृ स्वास्थ्य सुधारमा महिला स्वास्थ्य स्वयं सेविकाको (म.स्वा.से) भूमिका

अनुसन्धान कर्ताको नाम

सरिता पाण्डे

सुपरिवेक्षक

पदम सिम्खडा, इडविन भान तेजलिङ्गेन

सहभागी चिनारी पत्र

१. मैले यो अनुसन्धान सम्बन्धि **सहभागी जानकारी पत्र** पढे र यस सम्बन्धि प्रश्नहरु राखे । ☐

२. मेरो सहभागीता स्वेच्छिक हो र म कुनै पनि वेला यो अनुसन्धानमा भाग लिनबाट पछि हट्न सक्छु । ☐

३. मेरो विचारहरु मेरा व्यक्तिगत चिनारी बिना यस अनुसन्धान कर्ताहरुले प्रयोग गर्न पाउने छन् । मेरो नाम यस अनुसन्धानसंग जोडिने छैन । ☐

४. मेरा विचारहरु भविष्यमा यस अनुसन्धान सम्बन्धि अध्ययन तथा प्रकाशनमा प्रयोग गर्न म पूर्ण सहमत छु । ☐

५. म यो अनुसन्धानमा सहभागी हुन पूर्ण सहमत छु । ☐

सहभागीको नाम	मिती	हस्ताक्षर
अनुसन्धान कर्ताको नाम	मिती	हस्ताक्षर
सरिता पाण्डे		

सामूहिक छलफलका लागि आवश्यक चेकलीष्ट (महिला स्वास्थ्य स्वयं सेविका)

१. तपाइहरु कुन कुन काम गर्नहुन्छ ?

२. तपाइलाई आफ्नो काम गर्न कुनकुन (के) कुराले मद्दत पुर्‍याएको छ ?

३. के तपाई आफ्नो क्षेत्रको सम्पूर्ण घरहरुमा पुग्न सफल हुनु भएको छ ? यदि छैन भने के-कुराले तपाइलाई बाधा पुर्‍याएको छ ?

४. किन समुदायले तपाइको सेवालाई रुचाउँछन् ?
